# An Update on Pituitary Neuroendocrine Tumors Leading to Acromegaly and Gigantism

**DOI:** 10.3390/jcm10112254

**Published:** 2021-05-22

**Authors:** Sylvia L. Asa, Shereen Ezzat

**Affiliations:** 1 Department of Pathology, University Hospitals Cleveland Medical Center, Case Western Reserve University, Cleveland, OH 44106, USA; 2 Department of Medicine, University Health Network, University of Toronto, Toronto, ON M5G 2C4, Canada; shereen.ezzat@utoronto.ca

**Keywords:** growth hormone, acromegaly, gigantism, pituitary neuroendocrine tumor, hypothalamic gangliocytoma, ectopic GHRH

## Abstract

An excess of growth hormone (GH) results in accelerated growth and in childhood, the clinical manifestation is gigantism. When GH excess has its onset after epiphyseal fusion at puberty, the overgrowth of soft tissue and bone results in acromegaly. Persistent GH excess in gigantism also causes acromegalic features that become evident in the adult years. The causes of GH excess are primarily lesions in the pituitary, which is the main source of GH. In this review, we provide an update on the clinical, radiological and pathologic features of the various types of pituitary neuroendocrine tumors (PitNETs) that produce GH. These tumors are all derived from PIT1-lineage cells. Those composed of somatotrophs may be densely granulated, resembling normal somatotrophs, or sparsely granulated with unusual fibrous bodies. Those composed of mammosomatotrophs also produce prolactin; rare plurihormonal tumors composed of cells that resemble mammosomatotrophs also produce TSH. Some PitNETs are composed of immature PIT1-lineage cells that do not resemble differentiated somatotrophs, mammosomatotrophs, lactotroph or thyrotrophs; these tumors may cause GH excess. An unusual oncocytic PIT1-lineage tumor known as the acidophil stem cell tumor is predominantly a lactotroph tumor but may express GH. Immature PIT1-lineage cells that express variable amounts of hormones alone or in combination can sometimes cause GH excess. Unusual tumors that do not follow normal lineage differentiation may also secrete GH. Exceptional examples of acromegaly/gigantism are caused by sellar tumors composed of hypothalamic GHRH-producing neurons, alone or associated with a sparsely granulated somatotroph tumor. Each of these various tumors has distinct clinical, biochemical and radiological features. Data from careful studies based on morphologic subtyping indicate that morphologic classification has both prognostic and predictive value.

## 1. Introduction

Growth hormone (GH) is a 191 single chain amino acid polypeptide that signals through the GH receptor (GHR), a member of the cytokine receptor superfamily. Its primary effect is stimulation of insulin-like growth factor (IGF)-1 production, mainly in the liver, but it also has direct effects on almost every cell of the body. Together, GH and IGF-1 regulate cell growth and metabolism and are responsible for normal growth during development as well as the preservation of normal body composition in adulthood. GH regulates glucose and fatty acid metabolism. Other actions of GH include its impact on mental health and well-being, as well as cognitive functions, and it is implicated in immunomodulation [1].

GH is produced in the pituitary and its synthesis and secretion are regulated by stimulation and inhibition from the hypothalamus. The main stimulus is GH releasing hormone (GHRH), which is produced in the arcuate nucleus of the hypothalamus; the main inhibitor is somatostatin, which is produced in the paraventricular and infundibular nuclei of the hypothalamus.

GH has a short half-life (estimated to be approximately 3 min) and its secretion is pulsatile, complicating biochemical assessment. Circulating IGF-1 is more stable and represents an integrated measure of GH secretion [2].

This review outlines the features of GH excess and the various pathological entities that cause hypersecretion of this hormone.

## 2. Manifestations of GH Excess

Excess GH results in exaggerated growth. When it has its onset after epiphyseal fusion at puberty, the overgrowth of soft tissue and bone causes the features of acromegaly. Bone and soft tissue disfigurement include prominence of the forehead, enlargement of the nose, prognathism of the jaw, thickening of the tongue and lips, separation of the teeth, increases in the size of the hands and feet that lead to increased ring and shoe size, carpal tunnel syndrome and ultimately severe osteoarthritis [3]. The changes occur gradually, and due to the insidious nature of their development, they can go unrecognized for a long time. They are usually detected once found by someone who has not witnessed their gradual development, and often are identified when the patient is being investigated for manifestations of complications of the disease.

When the onset of GH excess is in childhood, the result is gigantism. The excessive growth of gigantism is readily diagnosed but even so, may be delayed. Persistent GH excess in gigantism also causes acromegalic features.

The additional signs and symptoms of GH excess are even more subtle than the external disfigurement. GH excess results in type 2 diabetes mellitus, hypertension and cardiomegaly, sleep apnea, deepening of the voice, thickening of the skin with excess sebaceous activity, sweating and body odor, the growth of skin tags, fatigue and muscle weakness [3]. Indirect effects of pituitary tumor growth and secretion include headaches, visual impairment, decreased libido, galactorrhea, and menstrual irregularities in women. Long term, GH and IGF-1 excess reduces life expectancy and can be associated with cancer development in multiple body sites [4,5,6].

The various manifestations differ depending on the cause of GH excess.

## 3. Causes of GH Excess

The most common cause of GH excess is a tumor of the pituitary, which is the main source of GH. Adenohypophysial cells that produce GH are members of the PIT1-lineage, since the transcription factor PIT1 is required for GH gene transcription. The normal cell types that produce GH are somatotrophs, which produce only GH, and mammosomatotrophs, which produce both GH and prolactin (PRL). Tumors of adenohypophysial neuroendocrine cells, known as adenomas, but more recently recognized as pituitary neuroendocrine tumors (PitNETs), include neoplastic proliferations of these cells but also of other unusual neoplastic cells that are not found in the normal gland. These are discussed in detail below.

A rare cause of pituitary-dependent GH excess is primary hyperplasia of somatotrophs or mammosomatotrophs; this is most common in cases of germline susceptibility, and has been reported in patients with McCune–Albright syndrome [7,8], X-linked acrogigantism [9,10] and Carney complex [11,12]). The distinction of hyperplasia from neoplasia is based on the reticulin pattern. Normal adenohypophysis has an acinar architecture in which admixtures of the various adenohypophysial hormone-producing cell types form acini with an intact reticulin network surrounding each acinus. In contrast, adenohypophysial cell neoplasms have a breakdown of the normal reticulin network. Pituitary hyperplasia is defined as a multifocal proliferation of a single cell population (in this case, either somatotrophs or mammosomatotrophs) causing expansion but not breakdown of the reticulin network [13]. Many examples of primary pituitary hyperplasia progress to neoplasia [8,9,10,11,12].

Rarely, GH excess is attributed to extra-pituitary lesions, mainly those that produce excess GH-releasing hormone (GHRH); these occur in the lung, pancreas, adrenal gland and occasionally in the hypothalamus, where they can mimic primary adenohypophysial lesions because they cause pituitary somatotroph hyperplasia. Exceptional ectopic GH production has been reported in the pancreas and teratomas.

## 4. Pituitary Neuroendocrine Tumors (PitNETs)

There are at least six distinct morphologic tumor types [13] and each has a different spectrum of clinical, biochemical and radiological features [14].

### 4.1. Densely Granulated Somatotroph Tumors

**Figure 1:**
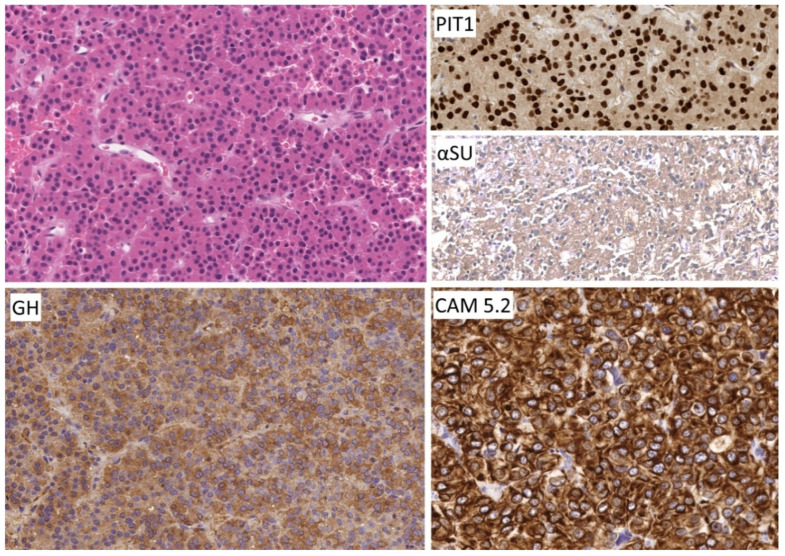
Morphology of densely granulated somatotroph tumor. The tumor cells with abundant acidophilic cytoplasm for solid nests and acini within a vascular stroma. The tumor cells exhibit strong PIT1 reactivity and diffuse cytoplasmic staining for GH as well as alpha subunit of glycoprotein hormones (αSU). Staining for keratins shows perinuclear positivity. No scale bar on the program used.

**Figure 2:**
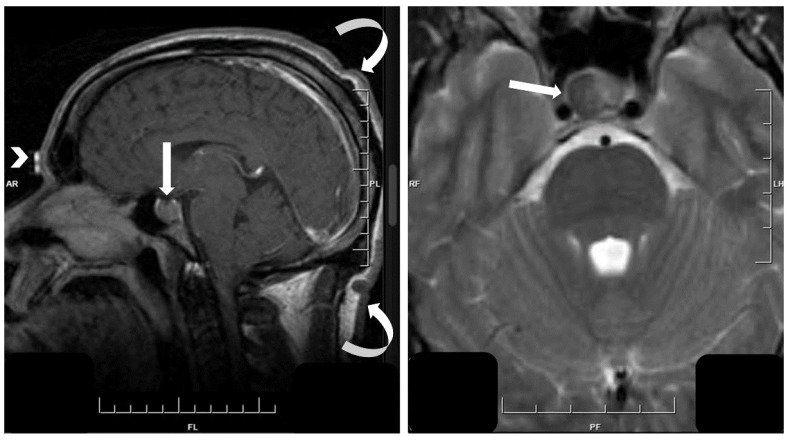
Magnetic resonance imaging of densely granulated somatotroph tumor. The sella turcica contains an intrasellar tumor (**left**, straight arrow); on T2-weighted imaging it is hypointense (**right**, straight arrow). Note the evidence of acromegaly in the thickened wavy skin (**left**, curved arrows) and frontal bossing (**left**, chevron).

Densely granulated somatotroph tumors are the classical lesions [15] that cause almost half of cases of GH excess [16]. These tumors are composed of cells that resemble normal somatotrophs (Figure 1). The tumor cells are strongly acidophilic on routine histology, because, as their name implies, they contain numerous secretory granules and on electron microscopy, are large and relatively homogenous with electron-dense contents. On immunohistochemistry, they have nuclear positivity for the transcription factor PIT1, and diffuse strong cytoplasmic staining for GH and alpha-subunits of glycoprotein hormones (αSU) [13], a feature of normal somatotrophs and explained by the high levels of cAMP that characterize these cells. Like normal somatotrophs, they have perinuclear accumulations of intermediate filaments that are keratin, and are identified by several keratin antibodies, including the CAM 5.2 antibody, the AE1/AE3 antibody cocktail and cytokeratin 18 [17]. Occasional tumors may have scattered cells that harbor aggregates of keratin that resemble fibrous bodies of the sparsely granulated somatotroph tumor discussed below, but these tumors, initially classified as “intermediate” [18], are now known to be clinically and metabolically similar to the classical densely granulated somatotroph tumors and therefore are no longer classified separately. Densely granulated somatotroph tumors are usually associated with florid clinical features and markedly elevated circulating GH and IGF-1 levels [19,20,21,22,23,24,25]. GH is usually the only functional hormone secreted by these tumors. Although they produce αSU [13], this is not usually measured in the circulation and little is known about its value in the clinical diagnosis and management of acromegaly. These tumors do not produce prolactin; patients with macrotumors may have hyperprolactinemia but it is generally under 200 ug/L and is attributed to the interruption of dopaminergic neurons in the pituitary stalk, releasing the normal gland from tonic inhibition. Patients with pure somatotroph tumors have been reported to be significantly more likely to have abnormal glucose metabolism than those with tumors that synthesize prolactin, a finding that is independent of GH/IGF-1 levels or tumor invasiveness and that appears to be independent of IGF-1 normalization [26]. The average age of diagnosis is around the age of 50 [22,24,25]. Imaging reveals a pituitary tumor that varies from an intrasellar microtumor (Figure 2) to a larger lesion with invasion laterally into the cavernous sinuses [24]. Upon administration of gadolinium, the normal gland, usually identified compressed around or beside the tumor, shows enhancement that is not seen in the tumor. The lesions are typically hypointense on T2-weighted imaging [27,28]. The florid acromegaly also gives rise to features that can be seen on MRI, including frontal bossing, a large tongue, and skin thickening that results in redundancy and waviness of the scalp.

### 4.2. Sparsely Granulated Somatotroph Tumors

**Figure 3:**
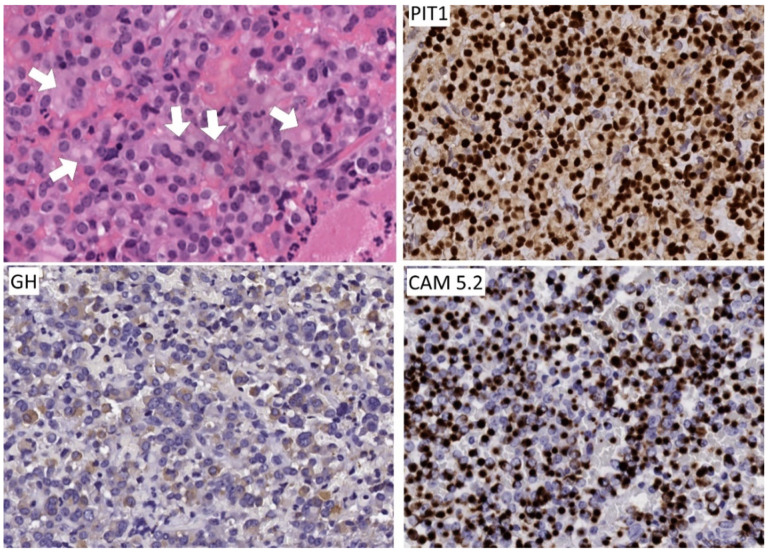
Morphology of sparsely granulated somatotroph tumor. The tumor cells are discohesive and pleomorphic with prominent cytoplasmic pale globules seen on H&E (**top left**, arrows). The tumor cell nuclei are intensely positive for PIT1 that highlights their pleomorphism. Staining for GH is weak and focal and can be easily missed. The keratin stain identifies the highly characteristic fibrous bodies that are found in the vast majority of tumor cells and are the hallmark of this tumor type.

**Figure 4:**
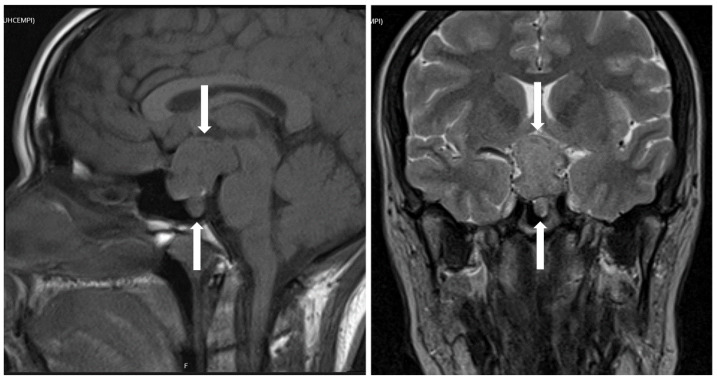
Magnetic resonance imaging of sparsely granulated somatotroph tumor. The pituitary tumor has expanded upwards to compress the optic chiasm and infiltrated downwards through the floor of the sella turcica into the sphenoid sinus (straight arrows, **right** and **left**). On T2 weighted imaging this lesion is hyperintense.

Sparsely granulated somatotroph tumors are also a common cause of GH excess [16]. These tumors (Figure 3) are composed of somatotrophs that express nuclear PIT1 and cytoplasmic GH but the GH positivity is usually weak and focal [13] and the tumor cells do not resemble normal somatotrophs. The discohesive cells are chromophobic on routine histology and have a prominent cytoplasmic inclusion that distorts the nucleus and corresponds to an aggregate of intermediate filaments on electron microscopy. This so-called “fibrous body” that occupies much of the tumor cell cytoplasm, trapping the few secretory granules and other organelles, is decorated by CAM 5.2, AE1/AE3 and CK18 antibodies [17] and represents the hallmark of this tumor type, being present in well over 70% of tumor cells. These tumors do not usually express any other pituitary hormones, including αSU. The discohesive nature of the tumor cells is usually obvious and is associated with the loss of the expression of E-cadherin [29,30,31]. Sparsely granulated somatotroph tumors are characterized by a more subtle clinical picture that may be easily overlooked, resulting in the mistaken clinical diagnosis of a “silent” somatotroph tumor [32,33]. True silent somatotroph tumors do occur but to be properly classified as such, they must be proven to not cause systemic GH or IGF-1 excess [34]. In contrast, these “quiet” tumors result in modest increases in circulating GH and IGF-1 levels that are typically lower than in their densely granulated counterparts [19,20,21,22,23,24,25]. Sparsely granulated somatotroph tumors do not make additional hormones, and if there is hyperprolactinemia, it is most likely to be due to stalk interruption (see above, Section 4.1). As indicated above, patients with sparsely granulated somatotroph tumors are also more likely to have abnormal glucose metabolism than those with tumors that secrete prolactin, discussed below [26]. The average age of onset is 10 years younger than patients with densely granulated tumors [22,24,25]. This may be because they grow faster and are more structurally aggressive but may also be due to the fact that they appear to be the hallmark of patients with germline AIP mutations, including the early onset giants [35]. There is also evidence of AIP downregulation in sporadic tumors of this type [36,37]. These tumors are typically invasive macrotumors with extrasellar invasion at the time of diagnosis (Figure 4) [18,23,24,25]; gadolinium enhancement identifies the residual nontumorous gland. They are typically hyperintense on T2-weighted magnetic imaging [27,28].

### 4.3. Mammosomatotroph Tumors

**Figure 5:**
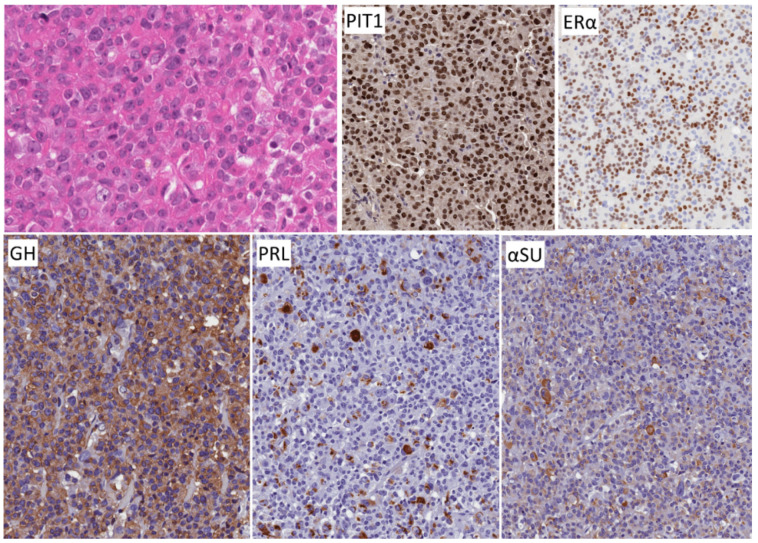
Morphology of mammosomatotroph tumor. These tumors resemble densely granulated somatotroph tumors (compare with Figure 1). They are composed of sheets or nests of strongly acidophilic cells that stain for PIT1, GH and αSU but in addition, they express nuclear ERα and have variable cytoplasmic positivity for prolactin (PRL).

**Figure 6:**
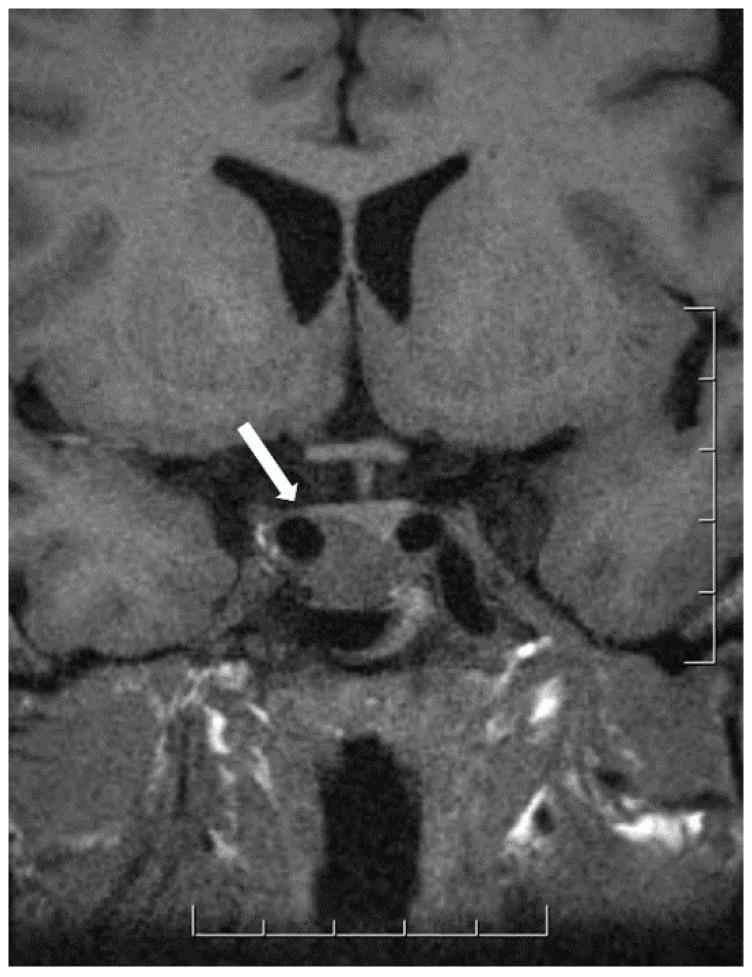
Magnetic resonance imaging of mammosomatotroph tumor. The sella turcica is enlarged and contains a tumor that compresses the normal gland upwards and to the left; the tumor infiltrates laterally to invade the right cavernous sinus (white arrow).

Mammosomatotroph tumors are morphologically and clinically very similar to densely granulated somatotroph tumors. They arise from mammosomatotrophs, normal cells that produce both GH and prolactin [38,39,40,41]; these cells are thought to be a fluid cell population that serves to transition somatotrophs to lactotrophs during pregnancy and lactation and revert back to somatotrophs when lactation concludes. They resemble somatotrophs in almost every way except that ultrastructurally, they have more pleomorphic secretory granules, and they exhibit misplaced exocytosis, which is a feature of prolactin secretion. Mammosomatotroph tumors (Figure 5) are composed of strongly acidophilic, densely granulated cells that express PIT1 but also the estrogen receptor alpha (ERα) and they have strong cytoplasmic GH reactivity and αSU with variable amounts of prolactin in most tumor cells. They have the perinuclear pattern of keratins that is seen in densely granulated somatotroph tumors. Clinically and biochemically, they are similar to densely granulated somatotroph tumors, with florid features and elevated GH and IGF-1 levels, but they also have manifestations of prolactin excess, including amenorrhea and decreased libido, due to prolactin production by the tumor. Patients with mammosomatotroph tumors are less likely to have abnormal glucose metabolism independent of GH/IGF-1 levels [26]. These tumors are sometimes found in younger patients [42,43] and are the characteristic feature of McCune–Albright syndrome [7,8,11], Carney complex [11,12] as well as the early onset X-linked acrogigantism known as X-LAG [9,10,44], where they may be associated with underlying hyperplasia. Like their densely granulated somatotroph counterparts, imaging reveals a pituitary tumor that varies from intrasellar to a lesion with invasion laterally into the cavernous sinuses (Figure 6) and lacks gadolinium enhancement. In one series, they were smaller and less invasive than pure GH-secreting tumors at diagnosis and therefore achieved better gross total resection [45]. The tumors are typically hypointense on T2-weighted magnetic imaging, rendering them difficult to distinguish radiographically from pure densely granulated somatotroph tumors, but they are clearly distinct from sparsely granulated somatotroph tumors clinically, radiologically, and morphologically.

### 4.4. Mature Plurihormonal Tumors of PIT1-Lineage

Mature plurihormonal tumors of PIT1-lineage are similar to mammosomatotroph tumors but also produce TSH [46]. These acidophilic tumors express PIT1, ERα as well as GATA3 [47]; the latter two may be focal and usually correspond with the expression of prolactin and TSH, respectively, whereas PIT1, GH and αSU are usually diffusely positive. The keratin profiles are usually diffuse and perinuclear. These patients may present with features of secondary hyperthyroidism. They are usually associated with florid acromegaly, very high levels of GH, IGF-1, prolactin and inappropriately normal TSH in the face of elevated T4 and T3 serum levels [48]. On imaging, they may appear as invasive macrotumors or, less often, intrasellar microtumors. They are hypointense on T2-weighted magnetic imaging, like densely granulated somatotroph and mammosomatotroph tumors.

### 4.5. Immature PIT1-Lineage Tumors

**Figure 7:**
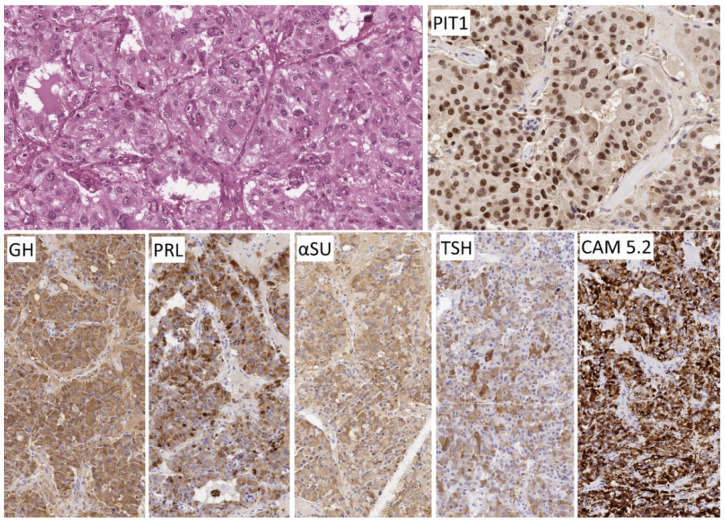
Morphology of immature PIT1-lineage tumor. The polygonal to spindle-shaped cells consistently express PIT1 that highlights the nuclear atypia and negatively staining intranuclear inclusions. Immunoreactivity for hormones and keratins is extremely variable as is the clinical presentation; this tumor that caused acromegaly and hyperthyroidism stains for GH and TSH, PRL and αSU. Keratin reactivity is intense as identified with CAM 5.2 and focal fibrous bodies are seen.

**Figure 8:**
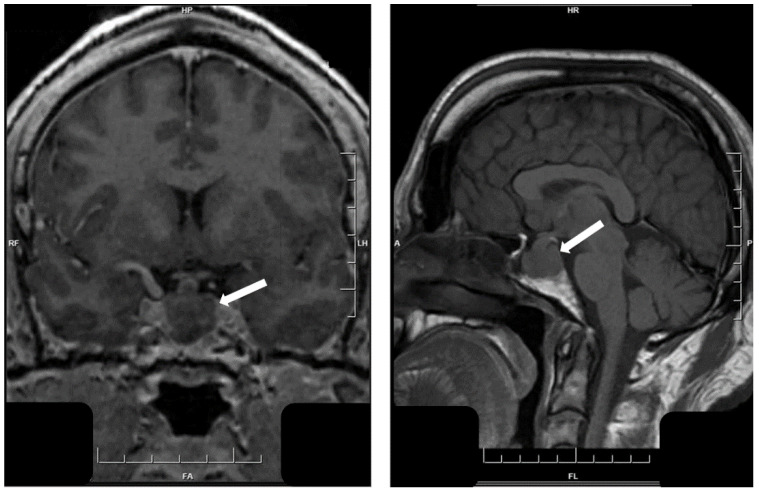
Magnetic resonance imaging of immature PIT1-lineage tumor. This large tumor invades laterally into both cavernous sinuses (arrow, **left**) extends upwards, forming a “snowman”-like mass herniating above the diaphragma sella (arrow, **right**).

Immature PIT1-lineage tumors are sometimes associated with acromegaly, but the clinical features are variable [49]. These tumors are composed of immature cells that all express PIT1 but lack the morphologic and immunohistochemical features of terminally differentiated cells of that lineage (Figure 7). While the cells are often more spindle-shaped, they may be polygonal. They all express PIT1, but the expression of ERα, GATA3, GH, prolactin and TSH is usually focal and variable. The keratin pattern also is variable, and the tumor cells may be negative. They may exhibit perinuclear keratin filaments, and they harbor cytoplasmic fibrous bodies. These tumors were initially classified as “silent subtype 3” tumors because they were initially thought to be variants of silent corticotroph tumors [50]. The clinical features are highly variable [49,51]. GH excess is a clinical feature of about 10–20% of patients with these tumors; TSH excess is more common, and hyperprolactinemia is often attributable to tumor production rather than the stalk effect. The age of onset is highly variable but a significant number of patients with these tumors are young, even under the age of 18 years, and a number of patients with these tumors have been diagnosed with multiple endocrine neoplasia type 1 [49,51]. On imaging, these are almost always large tumors (Figure 8) with extensive invasion, including into the cavernous sinuses but also into the clivus and inferiorly into the sphenoid sinus [49]. They are aggressive tumors that frequently require multiple surgeries, radiation, and additional systemic therapies [51].

### 4.6. Acidophil Stem Cell Tumors

**Figure 9:**
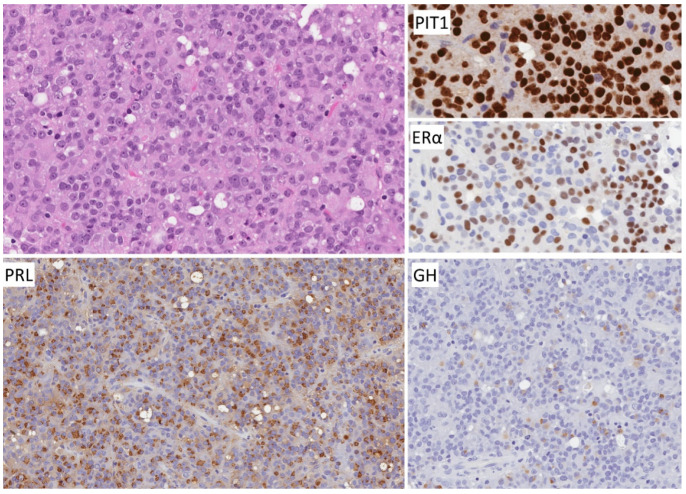
Morphology of acidophil stem cell tumor. This oncocytic tumor of PIT1 lineage has a characteristic histology with abundant, granular, pale acidophilic cytoplasm and scattered cytoplasmic vacuoles that represent dilated giant mitochondria. Tumor cells express nuclear PIT1 and ERα. There is extensive staining for PRL that shows a predominant juxtanuclear globular pattern of staining that corresponds to the Golgi region. Scattered tumor cells also express GH, explaining the development of variable GH excess in patients with these tumors.

**Figure 10:**
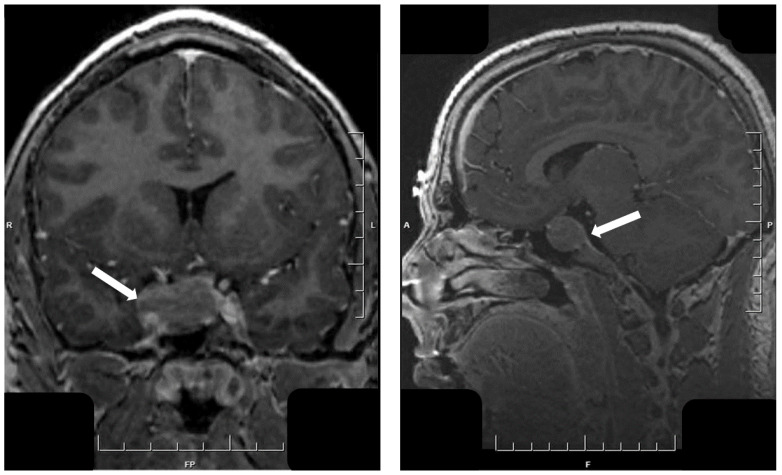
Magnetic resonance imaging of acidophil stem cell tumor. The large tumor that fills the sella turcica and extends into the right cavernous sinus (**left**, arrow) and characteristically downwards into the sphenoid sinus (**right**, arrow), was associated with hyperprolactinemia but not proportional to the tumor size; biochemical assessment documented mild elevation of IGF-1.

Acidophil stem cell tumors are rare lesions that have as their morphological hallmark intense oncocytic change with the formation of giant mitochondria that can be seen on routine histology [13] (Figure 9). These tumors are thought to be derived from a precursor of somatotrophs and lactotrophs; they express PIT1, ERα and prolactin, and they express GH focally. While somatotroph tumors virtually always have intense keratin positivity and may form fibrous bodies, lactotrophs may be only weakly positive or even negative [16]. It is not surprising, therefore, that acidophil stem cell tumors have variable keratin expression, but they may be more intensely positive than lactotroph tumors and may even form scattered fibrous bodies. The clinical presentation of these tumors mimics prolactinoma; however, closer examination will reveal that unlike lactotroph tumors, the degree of hyperprolactinemia, while greater than would be attributed to stalk compression, does not correlate with tumor size. This alone should raise a suspicion of this tumor type, but in addition, the patient may have a phenomenon referred to as “fugitive acromegaly” with very subtle clinical and fluctuating biochemical features [52,53,54]. On imaging, these are almost always large tumors (Figure 10) with invasion, into the cavernous sinuses and sometimes downwards into the sphenoid sinus. These tumors are rare and have not been widely studied for clinico–pathological features. They have been reported in children [55] and in patients with *SDHx* mutations [56].

### 4.7. Mixed Tumors with GH Production

**Figure 11:**
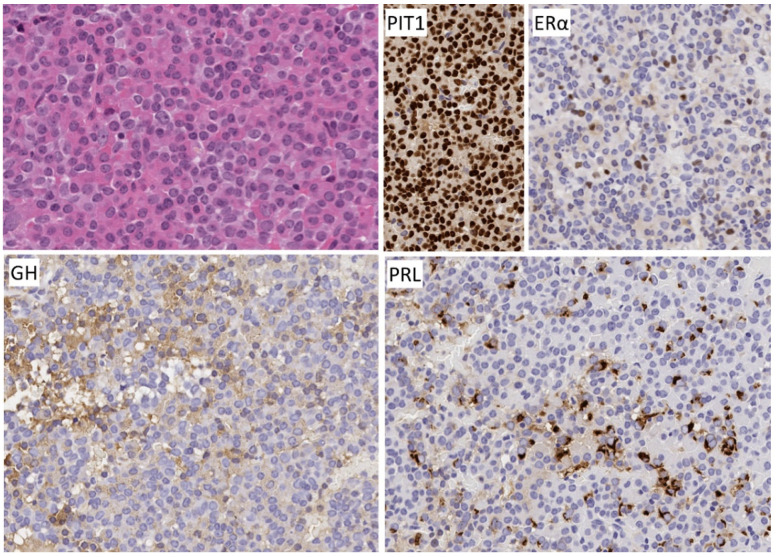
Morphology of mixed densely granulated somatotroph and sparsely granulated lactotroph tumor. This patient presented with acromegaly and had hyperprolactinemia. The tumor resembles a densely granulated somatotroph tumor but also has scattered cells with clear cytoplasm. There is diffuse nuclear positivity for PIT1, but only scattered cells express ERα. Positivity for GH is abundant but there are clearly negative cells that instead represent the clear cells which show juxtanuclear globular reactivity for PRL. The degree of GH excess in patients with these tumors is usually proportional to the GH producing cell mass as well as the type of GH-producing cells that may be densely granulated, as in this case, or sparsely granulated with fibrous bodies.

Mixed tumors with GH production are a highly variable group of tumors. The most common are mixed somatotroph–lactotroph tumors (Figure 11), but other synchronous multiple tumors occur [57] and the morphology is dependent on the cell types of the components. The presentation of GH excess varies with the proportion of the tumor that produces GH and the cytodifferentiation of that component (densely granulated vs. sparsely granulated somatotroph vs. mammosomatotroph) as well as the function and size of the other element with which it is mixed. In one series, mixed tumors were larger and more invasive with a higher degree of hyperprolactinemia than mammosomatotroph tumors [45].

### 4.8. Unusual Plurihormonal Tumors

Unusual plurihormonal tumors have also been reported in patients with GH excess. These monomorphous tumors have evidence of PIT1-lineage and other lineage commitments as well [58]. These are highly variable, and no consistent relationships can be drawn from these exceptionally rare lesions.

## 5. Hypothalamic Tumors

**Figure 12:**
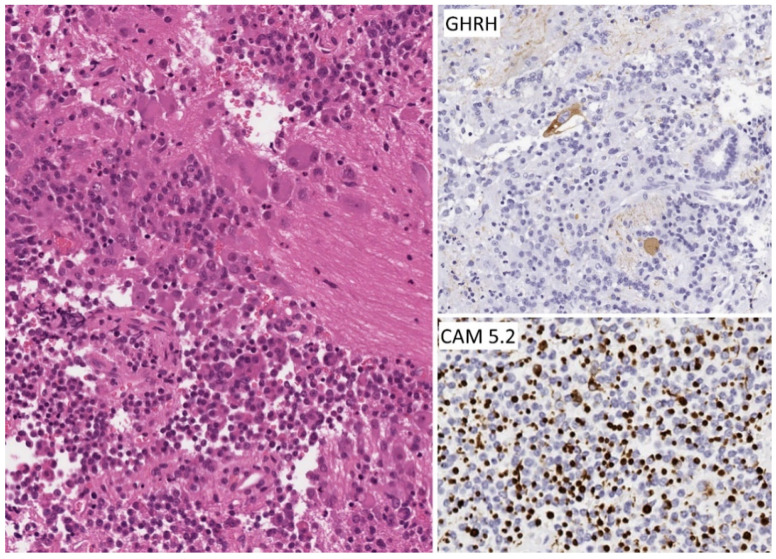
Morphology of hypothalamic gangliocytoma with sparsely granulated somatotroph tumor. This tumor is composed of two elements; the bulk of the lesion is a sparsely granulated somatotroph tumor similar to that shown in Figure 3, but there are scattered areas of neuropil with large neurons, known as “ganglion cells”, some of which are binucleate. The neurons contain immunoreactivity for GHRH, whereas the somatotrophs have fibrous bodies that are decorated by the CAM5.2 stain for keratins.

**Figure 13:**
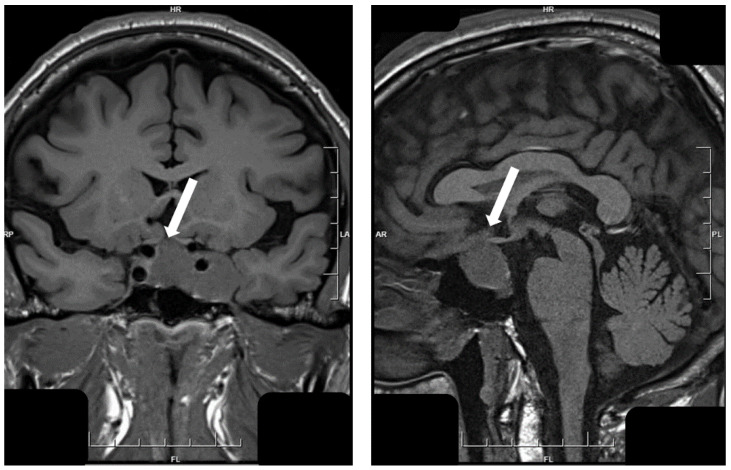
Magnetic resonance imaging of hypothalamic gangliocytoma with sparsely granulated somatotroph tumor. This tumor fills the sella turcica and the left cavernous sinus but also shows a thin attachment to the hypothalamus (white arrows).

Rare hypothalamic tumors composed of neurons can produce GHRH [59]. The majority have been *gangliocytomas*, tumors composed of magnocellular neurons of hypothalamic differentiation that have been reported as pure neuronal tumors [60] but are more often associated with a sparsely granulated somatotroph tumor [60,61,62,63] (Figure 12). These tumors occur at any age and give rise to significant acromegaly or even gigantism [60]. The details of the clinical and biochemical features are not well described and most have not been suspected to be a hypothalamic tumor prior to the pathology diagnosis. However, careful imaging can sometimes identify extension into the hypothalamus that provides a clue to this diagnosis (Figure 13). Neurocytomas are tumors composed of small neurons [64,65]; they only rarely occur in the hypothalamus and most often are associated with the syndrome of inappropriate diuresis due to production of vasopressin [66]; however, a *neurocytoma* has been reported as a rare cause of acromegaly in a single case [67].

## 6. Conclusions

GH excess represents a continuum of gigantism in childhood, progressing to acromegaly of adulthood. It is caused by neuroendocrine neoplasms, the vast majority arising in the sella turcica. The various pituitary neuroendocrine tumors that produce GH give rise to distinct clinical, biochemical and radiological findings.

The most typical situation involves florid acromegaly due to a densely granulated pituitary somatotroph tumor; however, this represents only about half of the cases of GH excess. Sparsely granulated somatotroph tumors give rise to less florid features of GH excess and tend to be larger masses that can be identified by their hyperintensity on T2-weighted imaging. Mammosomatotroph tumors resemble densely granulated somatotroph tumors in all aspects, but in addition, they express prolactin and give rise to significant hyperprolactinemia. Mature plurihormonal PIT1-lineage tumors are essentially mammosomatotroph neoplasms that also express TSH and may be associated with hyperthyroidism. Acidophil stem cell tumors and immature PIT1-lineage tumors are less well differentiated and have an extremely variable clinical presentation; not all patients have GH excess and when present, it is variable; these tumors are also associated with hyperprolactinemia and, in the case of the immature PIT1-lineage neoplasms, hyperthyroidism. Both of these variants tend to be more invasive and resistant to medical therapies. Unusual plurihormonal PitNETs that do not maintain adenohypophysial lineage fidelity can produce GH in association with other hormones such as ACTH or gonadotropins. To add to the complexity of these multiple tumor subtypes, there are also mixed PitNETs that can be composed of two or even three cell populations; in this circumstance, the clinical manifestations of GH excess depend on the cell type and the proportion of the GH-producing component. Another rare situation is a hypothalamic GHRH-producing gangliocytoma that may stimulate somatotroph hyperplasia but more often co-exists with a sparsely granulated somatotroph PitNET.

While the clinical presentations of PitNETs causing acromegaly can be fairly straightforward, many challenges are faced by patients with these disorders. The complexities arise from the spectrum of tumor cell diversity which give rise to distinct patterns of hormone production and tumor growth progression [68]. The underpinnings of this clinical spectrum have emerged from morphologic tumor classifications. Nevertheless, such classifications require refinement across broader clinical, biochemical, and radiographic paradigms. The importance of tumor distinctions rests in their predictive value, as has been shown in multiple studies [14]. It is hoped that further progress will better guide the development of next generation screening and management approaches to disorders of GH excess.
